# Supplementary material for: The Evaluation of Lipid-Lowering Treatment in Patients with Acute Coronary Syndrome in a Hungarian Invasive Centre in 2015, 2017, and during the COVID-19 Pandemic—The Comparison of the Achieved LDL-Cholesterol Values Calculated with Friedewald and Martin–Hopkins Methods
Source: J Clin Med. 2024 Jun 11;13(12):3398. doi: 10.3390/jcm13123398 (PMC11204367; doi:10.3390/jcm13123398)
Supplement: Supplementary file 1 [file jcm-13-03398-s001.zip › Supplementary Table S1_acs_r2.pdf]

Supplementary Table S1. Serum lipid parameters at the time of acute coronary syndrome, and 6 and 12 months after the index event during the COVID year.

	Baseline (index) event	6 months	12 months
Number of patients (n)	531	186	229
Total cholesterol			
(mmol/L, median [IQ-range])	4.92 (3.94-6.15)	3.59 (2.87-4.54)	3.56 (2.96-4.39)
% change from baseline		-27%	-28%
P value compared with baseline		<0.001	<0.001
LDL- cholesterol (Friedewald-formula)			
(mmol/L, median [IQ-range])	3.20 (2.30-4.19)	1.64 (1.09-2.30)	1.60 (1.19-2.27)
% change from baseline		-49%	-50%
p value compared with baseline		<0.001	<0.001
HDL- cholesterol			
(mmol/L, median [IQ-range])	1.03 (0.86-1.23)	1.08 (0.91-1.27)	1.08 (0.86-1.37)
% change from baseline		+5%	+5%
p value compared with baseline		NS	NS
Non-HDL- cholesterol			
(mmol/L, median [IQ-range])	4.00 (2.92-5.03)	2.53 (1.98-3.50)	2.50 (1.95-3.24)
% change from baseline		-37%	-37%
p value compared with baseline		0.001	<0.001
Triglyceride			
(mmol/L, median [IQ-range])	1.40 (0.90-2.09)	1.45 (1.11-1.98)	1.40 (0.98-1.91)
% change from baseline		+3%	0%
p value compared with baseline		NS	NS

Abbreviation: HDL, high-density lipoprotein; IQ-range, interquartile range; LDL, low-density lipoprotein; NS: non-significant. Notes: Serum total cholesterol and triglyceride (TG) concentrations were determined using an enzymatic, colorimetric method; in the case of HDL-C using a homogeneous, enzymatic method from same vendor due to the standard laboratory techniques. Non-HDL-C is calculated by subtracting HDL-C from total cholesterol. LDL-C was calculated by the Friedeward formula as detailed in the methods section.
