# Supplementary figures and images for: The Evaluation of Lipid-Lowering Treatment in Patients with Acute Coronary Syndrome in a Hungarian Invasive Centre in 2015, 2017, and during the COVID-19 Pandemic—The Comparison of the Achieved LDL-Cholesterol Values Calculated with Friedewald and Martin–Hopkins Methods
Source: J Clin Med. 2024 Jun 11;13(12):3398. doi: 10.3390/jcm13123398 (PMC11204367; doi:10.3390/jcm13123398)

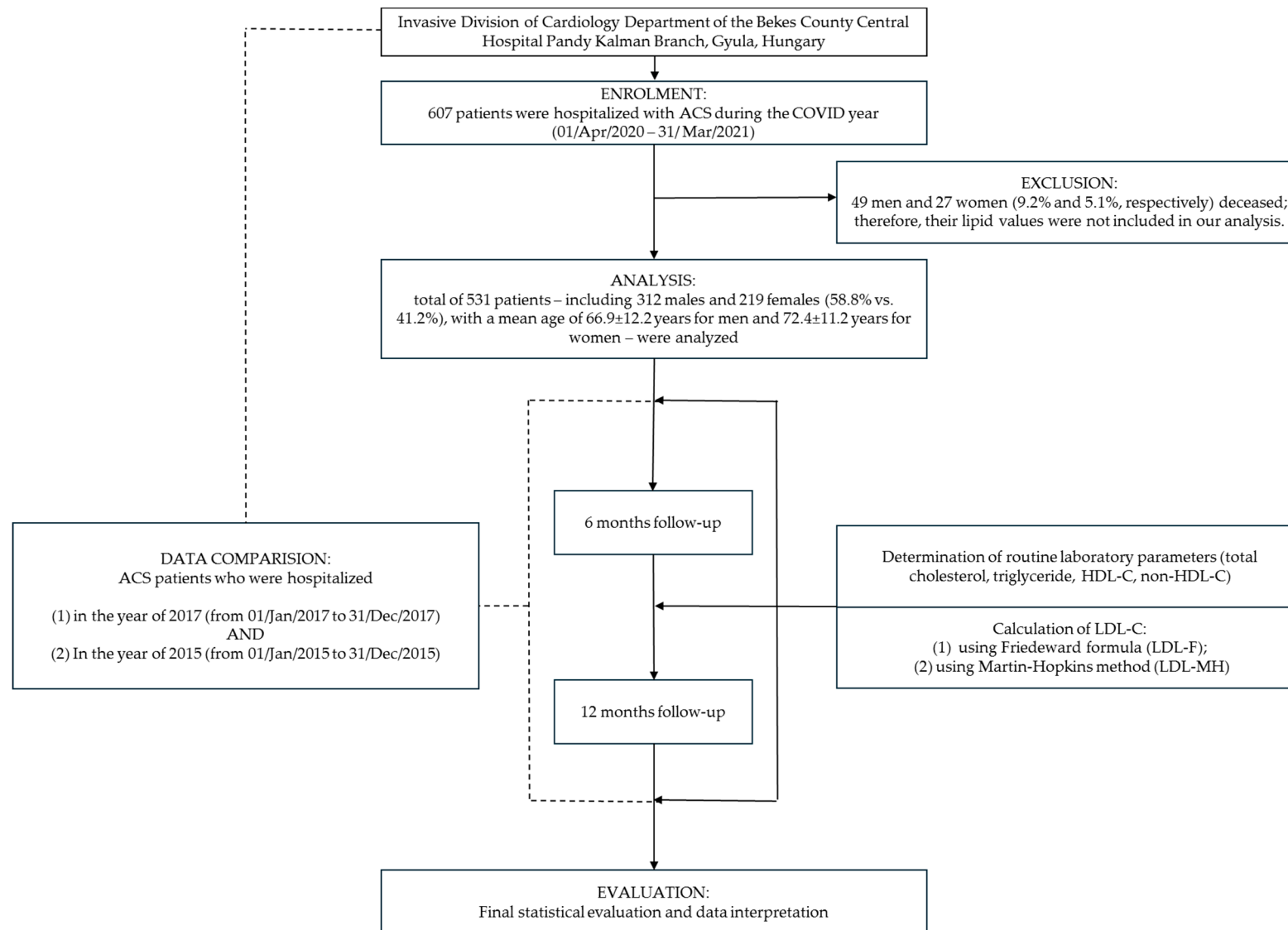

**Supplementary Figure S1.** Study design flowchart of enrolled subjects

Supplement: Supplementary file 1 [file jcm-13-03398-s001.zip › Supplementary Figure S1.pdf]
